# Hermansky‐Pudlak Syndrome: From Molecular Pathogenesis to Targeted Therapies

**DOI:** 10.1002/iub.70025

**Published:** 2025-05-19

**Authors:** Francesca Tondi, Roberta Annamaria Cirsmaru, Chiara Conti, Antonia Follenzi, Paolo Gresele, Cristina Olgasi, Loredana Bury

**Affiliations:** ^1^ Department of Medicine and Surgery, Section of Internal and Cardiovascular Medicine University of Perugia Perugia Italy; ^2^ Department of Health Sciences, School of Medicine University of Piemonte Orientale Novara Italy; ^3^ Dipartimento Attività Integrate Ricerca Innovazione Azienda Ospedaliero‐Universitaria SS. Antonio e Biagio e C. Arrigo Alessandria Italy; ^4^ Department of Translational Medicine, School of Medicine University of Piemonte Orientale Novara Italy

## Abstract

Hermansky‐Pudlak syndrome (HPS) is a rare inherited disorder caused by defects in lysosome‐related organelles (LROs) in various tissues, including platelets, melanocytes, and endothelial cells. Key features of HPS include oculocutaneous albinism, bleeding tendency, and, in some cases, pulmonary fibrosis, granulomatous colitis, and immunodeficiency. The condition is linked to mutations in 11 genes involved in the formation of LROs. Currently, treatment options for HPS are limited and often ineffective. Though cell and gene therapies have been explored for melanosomes and epithelial cells, there is limited knowledge about their application to platelets and endothelial cells. Understanding the detailed mechanisms of HPS pathogenesis is crucial, and using induced pluripotent stem cell (iPSC) models may provide valuable insights into the disease's molecular processes, aiding the development of new treatments. In this review, we will focus on the genetics and molecular mechanisms of HPS, on its clinical manifestations and current therapeutic approaches, highlighting the need for further research into the disease mechanisms and potential innovative therapies.

## 1 Introduction

Hermansky‐Pudlak syndrome (HPS) is a rare inherited disorder characterized by a combination of oculocutaneous albinism and a bleeding tendency, with some patients also experiencing pulmonary fibrosis, granulomatous colitis, immune deficiency, and neurodegeneration [1]. HPS has been reported across various ethnic groups worldwide, including Caucasians, Europeans, Asians, Puerto Ricans, and non‐Puerto Rican Hispanics [2]. The global prevalence of HPS has been estimated to be between 1 in 500,000 and 1 in 1,000,000; however, the exact frequency of HPS remains unknown [2]. Due to a founder effect, Puerto Rico has the highest prevalence of HPS‐1 (1:1820) accounting for 50% of all cases worldwide [2]. The HPS Network Inc., a nonprofit organization, has registered approximately 1200 HPS individuals worldwide [2].

HPS is caused by variants in 11 different genes, all coding for proteins playing a role in the biogenesis of lysosome‐related organelles (LROs), leading to defects in cellular processes like protein trafficking and organelle function. There are several subtypes of HPS, each linked to a mutation in a specific gene, but all resulting in similar clinical features [2]. These mutations lead to defects or loss of function in the proteins involved in the biogenesis of lysosome‐related organelle complexes (BLOC‐1, BLOC‐2, or BLOC‐3), as well as the adaptor protein 3 (AP‐3) complex [3]. The most severe subtype is HPS‐1, in which the gene variants affect the HPS1 protein that interacts with HPS4 to form the BLOC‐3 complex. Individuals with HPS have abnormal melanosome formation, which causes the characteristic lack of pigmentation of the skin, hair, and eyes. The bleeding tendency, due to defects in platelet function, is variable, while pulmonary fibrosis, particularly frequent in HPS‐1, is a significant cause of morbidity and mortality. Currently, there is no cure for HPS, and its management typically focuses on symptomatic treatment, with lung transplantation being the only therapeutic option in case of advanced pulmonary fibrosis. One aspect hindering the development of a cure is the lack of in‐depth knowledge of the molecular mechanisms of the disease. In fact, to date, only a few studies have attempted to unveil the exact causes of LROs dysfunction [4]. This review will focus on the genetic and molecular mechanisms, and on the clinical manifestations of HPS. Current treatment options and future possible cell‐ and gene‐therapy approaches will also be explored.

## 2 Genetics and Diagnosis

### 2.1 HPS Subtypes and Mutations

In 1996 the *HPS1* gene was first identified as the site of the causative variant of HPS‐1 [5]. Thereafter, other 10 HPS genes and their protein counterparts have been identified as the sites of variants associated with the HPS‐2 to HPS‐11 subtypes [6]. The molecular function of the HPS genes is driven by different HPS protein‐associated complexes (HPACs): Adaptor Protein‐3 (AP‐3), Biogenesis of Lysosome‐Related Organelles Complex (BLOC)‐3, BLOC‐2, and BLOC‐1. HPACs are involved in the biogenesis of lysosome‐related organelles (LROs) through the regulation of endo‐lysosomal trafficking [7, 8, 9]. LROs are a diverse group of membrane‐bound intracellular compartments that share some features with lysosomes but have specialized functions tailored to specific cell types. They are involved in processes such as storage, secretion, and immune responses. Examples include melanosomes in pigment cells, Weibel‐Palade bodies (WPBs) in endothelial cells, and dense granules in platelets. The AP‐3 complex consists of 4 subunits and includes the protein products of *AP3B1*, which is mutated in HPS‐2, and *AP3D1*, mutated in HPS‐10. BLOC‐3 consists of the HPS1 and HPS4 proteins, defective in the subtypes HPS‐1 and HPS‐4, respectively. BLOC‐2 consists of the proteins HPS3, HPS5, and HPS6, and its deficiency causes subtypes HPS‐3, HPS‐5, and HPS‐6. BLOC‐1 consists of 8 subunits: DTNBP1 (BLOC1S8), BLOC1S4, BLOC1S6, BLOC1S5, Snapin (BLOC1S7), BLOC1S3, BLOC1S6, and BLOC1S5 [10]. DTNBP1, BLOC1S3, BLOC1S6, and BLOC1S5 are defective in HPS‐7, HPS‐8, HPS‐9, and HPS‐11, respectively [1] (Table 1).

**TABLE 1:** Hermansky‐Pudlak Syndrome subtypes, associated genes and protein complexes.

HPS subtype (phenotype MIM number)	Gene name (alternative names)	Protein complex	Human locus
HPS‐1 (203300)	*HPS1 (BLOC3S1)*	BLOC‐3	10q24.2
HPS‐2 (608233)	*AP3B1 (ADTB3)*	AP‐3	5q14.1
HPS‐3 (614072)	*HPS3 (BLOC2S1)*	BLOC‐2	3q24
HPS‐4 (614073)	*HPS4 (BLOC3S2)*	BLOC‐3	22q12.1
HPS‐5 (614074)	*HPS5 (BLOC2S2)*	BLOC‐2	11p15.1
HPS‐6 (614075)	*HPS6 (BLOC2S3)*	BLOC‐2	10q24.32
HPS‐7 (614076)	*DTNBP1 (HPS7, BLOC1S8)*	BLOC‐1	6p22.3
HPS‐8 (614077)	*BLOC1S3 (HPS8, RP, BLOS3)*	BLOC‐1	19q13.32
HPS‐9 (614171)	*BLOC1S6 (HPS9, PLDN, BLOS6)*	BLOC‐1	15q21.1
HPS‐10 (617050)	*AP3D1 (HPS10, ADTD)*	AP‐3	19p13.3
HPS‐11 (619172)	*BLOC1S5 (BLOS5, MUTED)*	BLOC‐1	6p24.3

### 2.2 Diagnosis and Genetic Testing

Clinical suspicion of HPS should arise in the presence of oculocutaneous albinism, bleeding diathesis, and in some cases, pulmonary fibrosis, immunodeficiency, or granulomatous colitis, particularly in individuals with relevant ethnic or familial backgrounds.

Clinical diagnosis of HPS involves a comprehensive evaluation of patients presenting with oculocutaneous albinism, ocular abnormalities, and bleeding diathesis. Cutaneous albinism is revealed by the finding of hypopigmentation of the skin and hair on physical examination, especially when compared to non‐affected family members. Cutaneous albinism is associated with ocular albinism; however, ocular albinism may be present also in patients with normally pigmented skin coloration. Ocular albinism is not always immediately apparent with a very light eye color; thus, it must be diagnosed by an ophthalmologist revealing reduced iris pigmentation at iris transillumination, reduced retinal pigment on fundoscopic examination, and foveal hypoplasia. Ocular abnormalities include nystagmus, photophobia, and strabismus, associated with a significant reduction in visual acuity [11].

Laboratory tests typically show normal platelet count and normal coagulation parameters but impaired secondary aggregation at light transmission aggregometry. Transmission electron microscopy (TEM), in particular the simplified technique of “whole mount”‐TEM, is considered the gold standard for the assessment of δ‐granule content in platelets [12, 13]. However, TEM can be carried out by few centers, and other tests for the assessment of δ‐granule content have been developed.

When platelets are exposed to ^3^H‐labeled serotonin (5‐HT), they readily absorb it into their stored 5‐HT pool, ready to be released. The proportion of 5‐HT released during activation can be determined by comparing agonist‐stimulated release with the total 5‐HT absorbed. This assay not only reveals defects in granule biogenesis when platelets fail to take up 5‐HT, but it also measures δ‐granule secretion. While highly sensitive and specific, making it the gold standard for δ‐granule secretion, its use is now limited due to costs and regulatory restrictions associated with radiolabeled isotope use. An alternative to the radiolabeled 5‐HT assay is o‐phthalaldehyde (OPT), which binds to 5‐HT and can be detected by fluorimetry. Other tests, also very little used, are the assessment of the release of 5‐HT by flow cytometry after specific intracytoplasmic staining with a fluorescent anti‐serotonin antibody, by ELISA, HPLC, or mass spectrometry [14].

A simple approach uses the green‐fluorescent marker mepacrine, which acts as an indirect marker of 5‐HT content and release as it utilizes the same transporter as 5‐HT to enter δ‐granules [15]. Mepacrine is rapidly taken up by δ‐granules and then is released upon platelet activation. Mepacrine uptake and release can be assessed by flow cytometry, requiring minimal blood volume; however, the method lacks standardization, leading to variability in results [15]. δ‐granule secretion can be evaluated by flow cytometry also using granular markers such as CD63 and LAMP‐2 (CD107b), which are expressed on the platelet surface following activation. However, these markers are expressed also in lysosomes; thus, they are not specific in the diagnostic setting [14]. On the contrary, assessment of CD63, LAMP‐1, and LAMP‐2 by immunofluorescence is a validated diagnostic tool for the diagnostic screening of HPS [16].

Several methods are available for measuring ATP and ADP levels, including plate‐based end‐point assays, HPLC, and lumiaggregometry, the latter being the most widely used approach. Lumiaggregometry employs a bioluminescent reaction catalyzed by firefly luciferase and represents a modification of traditional light transmission aggregometry which allows the measurement of platelet aggregation in combination with the bioluminescent measurement of ATP release and is a commonly used alternative to the radioactive serotonin incorporation/release assay. Lumiaggregometry is considered sensitive enough and specific when using strong agonists but not with weak agonists [13].

HPS diagnosis is confirmed by molecular genetic testing through the identification of pathogenic variants in one of the genes associated with HPS. Genetic testing approaches include either Sanger sequencing or next‐generation sequencing (NGS). Sanger sequencing is often employed when a specific mutation is suspected, such as in populations with a high prevalence of HPS, like Puerto Rican individuals, where the g.339_4260del3904 deletion in *HPS3* is quite common [17]. Sanger sequencing is, however, time‐consuming and costly, while NGS, on the other hand, allows for the comprehensive screening of multiple genes simultaneously, identifying rare variants or novel mutations across the 11 known HPS subtypes, being the technique of choice for genetic analysis of HPS. Identifying the specific HPS subtype is critical because each subtype is associated with different complications, as mentioned above. In rare cases in which a genetic variant in one of the 11 HPS genes is missed by NGS, but a clear laboratory and clinical phenotype suggests HPS, long‐read sequencing approaches can be a useful alternative [18].

HPS shares several clinical features with other syndromic disorders, making differential diagnosis important. Chediak‐Higashi syndrome (CHS), due to a defect in the *LYST* gene, is characterized, like HPS, by oculocutaneous albinism and a bleeding diathesis due to platelet storage pool deficiency. However, CHS also presents recurrent infections, immune dysregulation, and typical giant lysosomal granules in various cell types, a feature absent in HPS. Griscelli syndrome also features albinism and immunodeficiency, but it can be distinguished by the presence of large, abnormal melanosomes in skin biopsy samples. δ storage pool disease (δ‐SPD) presents with a similar platelet δ granule deficiency and bleeding tendency but lacks the syndromic features of HPS [17].

## 3 Molecular Mechanisms of Disease

### 3.1 Lysosomal‐Related Organelle Dysfunction (Melanocytes, Platelets, Alveolar Cells)

LROs are distinct cellular compartments characterized by unique cell type‐specific properties, such as composition and morphology, and exhibit similar features to endosomes and lysosomes [19]. The principal LROs are melanosomes in melanocytes, platelet‐dense granules, WPBs in endothelial cells, lamellar bodies (LBs) in alveolar type 2 (AT2) cells, and lytic granules in natural killer (NK) cells and cytotoxic T lymphocytes (CTLs). These organelles play a pivotal role in several biological processes, including hemostasis, pigmentation, immunity, and lung plasticity [20]. Their regulation is critical as they take part in sustaining fundamental cellular functions, and mutations in genes involved in LROs biogenesis and trafficking can disrupt the highly coordinated process of storage and control of the secretion of materials essential for tissue‐specific functions, resulting in the various pathological manifestations typical of HPS. Oculocutaneous albinism and bleeding tendency, present in all HPS patients, can be attributed to defective melanosomes, which lead to hypopigmentation of skin and hair, and to platelet dense granules, which lead to defective platelet function and abnormalities in endothelial WPBs possibly contributing to bleeding. In addition, other HPS subtypes are characterized by the concomitant presence of defects in other LROs, such as in AT2 cells, which contribute to severe pulmonary complications due to impairments in LBs that hamper surfactant production and secretion. Moreover, LROs are involved in immune responses, where deficiencies can disrupt the storage and release of critical immunomodulatory molecules.

### 3.2 Role of HPS Proteins in Vesicle Trafficking

**FIGURE 1:**
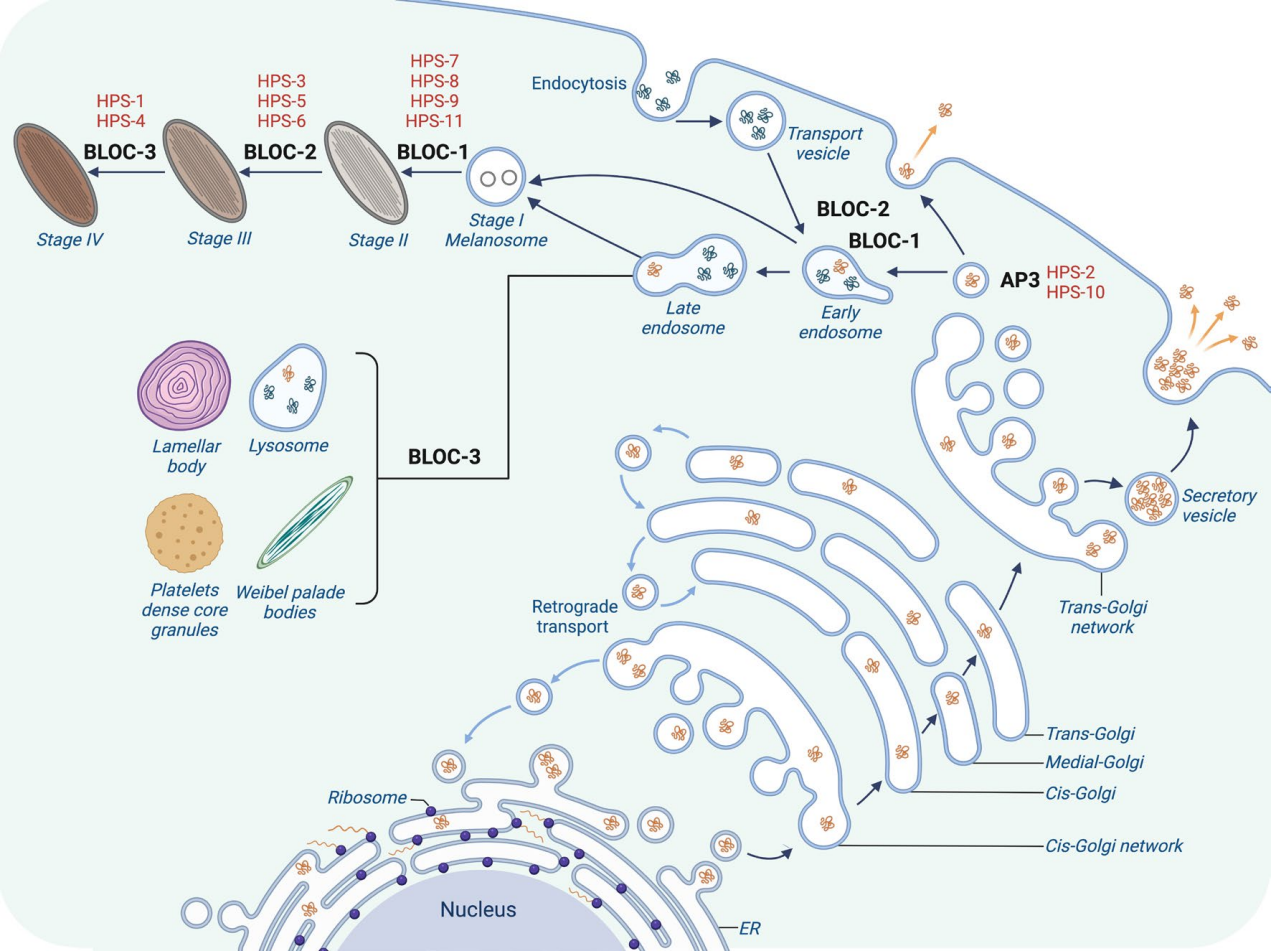
Representation of BLOC complexes involved in HPS, with their subunits and their role in the biogenesis of LROs. Created in BioRender. https://BioRender.com/k31r516.

The proteins encoded by the HPS genes function as part of multi‐subunit protein complexes that regulate intracellular trafficking, vesicle formation, and protein sorting to maintain cellular homeostasis (Figure 1). These proteins include AP3 and the BLOC‐1,‐2, and‐3, and defects in those protein complexes are associated with HPS subtypes. AP3 is a heterotetrameric complex composed of subunits δ, β3, μ3, and *σ*3. In mammals, β3, μ3, and *σ*3 subunits exist in two different isoforms (A and B) encoded by distinct genes contributing to the functional diversity of the protein [21, 22]. Based on the subunit composition, it is possible to distinguish a ubiquitous or tissue‐specific complex functionality: δ, β3A, μ3A, and *σ*3A/B (AP‐3A) are expressed in all tissues [23], whereas δ, β3B, μ3B, and *σ*3A/B (AP‐3B) subunits are mainly neuronal‐specific [24, 25, 26]. AP‐3 is essential for the transport of the cargo from the trans‐Golgi network (TGN) and/or early endosomes to lysosomes or LROs. The recruitment of the AP‐3 complex to membrane‐bound vesicles is mediated by a small GTPase known as ADP‐ribosylation factor 1 (Arf1) which promotes the formation of vesicles and stabilizes the AP‐3 complex during cargo recognition [27]. The interaction between AP‐3 and cargos is based on tyrosine and dileucine motifs‐binding sites, which facilitate protein sorting within the cell [28, 29]. Defects of the AP‐3 complex in humans lead to abnormalities in melanosomes [30], platelet dense granules, and defective WPBs maturation [31] and failure in the immune response, such as impaired trafficking of granules in CTLs [32] or toll‐like receptors (TLR) to phagosomes [33]. BLOC‐1 is an octamer [34, 35] with a multifunctional role in cellular processes. In melanocytes, BLOC‐1 is involved in the formation of tubular transport carriers, elongated structures that arise from early endosome membranes of melanosomes [36]. Thus, it is essential for the transport of protein cargos in the different stages of melanin synthesis, such as tyrosinase‐related protein‐1 (TYRP1) [37], oculocutaneous albinism type 2 (OCA2) protein [38], and the copper transporting ATPase (ATP7A) [39]. BLOC‐1 collaborates with cytoskeletal components, including the kinesin‐3 motor KIF13A, to stabilize and elongate transport tubules [40]. This interaction is modulated by the small GTPase Rab22A [41] and by SNARE proteins, such as syntaxin13 [42], important for membrane fusion [43]. While BLOC‐1 is predominantly associated with cargo delivery to melanosomes, its functions extend to transferrin recycling, lysosomal degradation, trafficking of epidermal growth factor receptor (EGFR) [44] and of synaptic vesicles in neurons [45], highlighting its versatile functions across different cell types. BLOC‐2 consists of three subunits [46]; it is involved in cargo delivery and in the direction of the tubular transport carrier to WPBs [47] and, together with BLOC‐1, to melanosomes. BLOC‐3 is a guanine nucleotide exchange factor (GEF) for Rab32/38, important for cargo delivery from early endosomes to melanosomes [48] and can also interact with Rab9a in the formation of melanosomes [49]. However, BLOC‐2 and 3 are still under investigation for their role in the pathogenesis of HPS.

### 3.3 Melanogenesis and Oculocutaneous Albinism

Melanosomes are large LROs (~500 nm in diameter) localized in melanocytes and retinal pigment epithelial cells, which are the site for the synthesis and storage of melanin, involved in the pigmentation of hair, skin, and eyes [50]. Melanin and melanosome biogenesis is known as melanogenesis, for which several biological and biochemical reactions are required. Melanosome maturation involves four stages (I‐IV), each characterized by a progressive structural and functional change due to the acquisition of protein cargo sorted throughout biogenesis. During stages I and II, melanosomes lack melanin and are thus called pre‐melanosomes, while at stage III, melanin synthesis starts due to the delivery of tyrosinase and tyrosinase‐related proteins (TYR, TYRP1, OCA2, and ATP7A) from early endosomes and, finally, in stage IV, melanosomes contain enough melanin to be transferred [51, 52]. The biogenesis and motility of stage I melanosome‐derived vesicular carriers are regulated by various trafficking components, including protein complexes that are affected by mutations in HPS. The vesicular route requires the AP‐3 complex, which is involved in the transport of TYR from early endosomes to stage III melanosomes, whereas the delivery of TYRP1, OCA2, and ATP7A is promoted by the tubular route. This process is characterized by the extension of the early endosome membrane driven by the interaction between BLOC‐1, Rab22A, and microtubule‐associated proteins such as KIF13A, and coordinates with other proteins, such as Annexin A2, required for the activation of ARP2/3‐dependent actin filaments leading to a further stabilization of the tubule formation. Therefore, mutations in BLOC‐1 result in the destabilization of the entire complex and a decrease in pigmentation, typical of HPS. BLOC‐2 is acting downstream of BLOC‐1 by directing the tubular endosomal transport carrier to stage III melanosomes, probably through the association with Rab22A, Rab32, and Rab38 [52, 53]. The delivery of cargo during the maturation of melanosomes is achieved via membrane trafficking through the regulation of intracellular membrane fusion by SNARE [54]. Bowman et al. showed that BLOC‐1 and AP‐3 coordinate the sorting of syntaxin‐13 and VAMP7, a vSNARE protein, through tubule transport carriers from early endosomes to mature melanosomes [42]. Moreover, VAMP7 is recycled from mature melanosomes by BLOC‐3 and Rab32/38‐dependent mechanisms [55]. Mutations in these complexes, typical of HPS, impair cargo sorting and delivery, causing the defective melanin synthesis and hypopigmentation characteristic of oculocutaneous albinism. However, further studies are needed to shed light on the trafficking pathway, to fully unravel the mechanisms of biogenesis of melanosomes, and finally to identify a therapeutic approach for the treatment of the diseases associated with melanin defects.

### 3.4 Platelet Dysfunction and Bleeding

A human platelet contains three to eight dense granules, or δ granules, 200–300 nm in diameter. Dense granules, with a luminal pH of 6.1, belong to the family of LROs and store small molecules at high concentrations (high mM range), like serotonin, ADP, ATP, Ca^2+^, pyrophosphate (PPi), and polyphosphates of 70–75 phosphate units (polyP), as well as Mg^2+^ and K^+^ [56].

Although the biogenesis of δ granules has been assumed to be similar to other LROs, such as melanosomes, it is poorly understood. It has been hypothesized that δ granules originate from megakaryocyte endosomes, not from the TGN, which are then transferred to platelets [56, 57]. The molecules contained in δ granules are transported from the megakaryocyte and platelet cytosol into the δ granule lumen.

Upon δ‐granule exocytosis, the released ADP and serotonin act as platelet agonists and play an important autocrine and paracrine role in amplifying platelet activation and aggregation [58]. PolyP released from platelet δ‐granules conglomerates into nanoparticles that accumulate on the platelet surface and promote FXII activation [59]. Additionally, polyP can enhance thrombin generation by favoring thrombin‐mediated activation of FXI and FV [60]. It is thus clear that the δ‐granule deficiency provokes a reduced secretion of platelet agonists, a defective second wave of aggregation, and impairment of the platelet contribution to blood clotting activation and, thus, bleeding [56].

### 3.5 Endothelial Cells and Bleeding

Endothelial cells (ECs) are a monolayer of cells lining blood vessels that regulate vascular homeostasis, immune response, and angiogenesis. A specialized storage organelle of ECs, WPBs, can have a role in their vascular functions, storing molecules, such as von Willebrand factor (VWF) or angiogenic mediators [31, 61]. Indeed, WPBs can be rapidly mobilized to the cell surface in response to various stimuli, such as injury or inflammation, contributing to the regulation of blood coagulation and vascular permeability. The dynamic release of proteins from WPBs plays an essential role in the EC's ability to respond to vascular injury, facilitating wound healing and controlling blood flow and clotting activation in response to physiological and pathological stimuli; thus, an impairment in WPBs can result in ECs dysfunction. Moreover, an alteration of WPBs has been shown in HPS‐6 deficient mice and may play a role in the increased bleeding tendency of patients [62].

Biogenesis of WBPs is strictly related to VWF maturation and consists of a multi‐step process. After the synthesis and first post‐translational modification, pro‐VWF dimers relocate from the endoplasmic reticulum (ER) to the TGN through GBF1, a guanine nucleotide exchange factor (GEF) [63]. Here, additional modifications occur: pro‐VWF dimers organize in multimers, condense into tubules, and pack in TGN‐associated WPBs [64, 65] thanks to the acidic pH within the TGN [66]. Recent evidence has shown that BLOC‐2 plays a role in the maintenance of the acidic environment of WPBs by sorting the v‐ATPase complex subunit ATP6V0D1, a proton pump responsible for acidifying intracellular compartments, to WPBs, further highlighting the crucial role of HPS proteins in WPBs biogenesis [67]. Once the multimerized tubular VWF is fully packed, the nascent WPBs vesicles arise from the TGN in an AP‐1/clathrin‐dependent process [68, 69], which is responsible also for the size and structure of WPBs. Mutations in the VWF gene, leading to the inherited bleeding disorder von Willebrand disease (VWD), lead to abnormal WPBs formation due to an impairment of VWF maturation [70].

Immature WPBs acquire additional protein cargos, such as CD63 and the SNARE protein VAMP8, from the endosomal compartment through an AP‐3‐dependent pathway [71]. Deficiency in the AP‐3 complex is associated with HPS2. Recently, endothelial colony‐forming cells (ECFC) isolated from an HPS‐2 patient and CRISPR‐Cas9‐engineered ECs with knocked‐down HPS2 were used as a model to study the impaired mechanism of maturation of WPBs and the subsequent impaired exocytosis, suggesting for the first time a link between impaired endothelial cell function and bleeding due to impaired VWF secretion from WPBs, a mechanism crucial for platelet adhesion during primary hemostasis. Indeed, AP‐3 dysfunction resulted in CD63‐trafficking failure with consequent altered WPBs maturation and failed recruitment of VAMP8, which is involved in WPBs exocytosis [72]. Evidence suggests that BLOC‐2 is also involved in the delivery of CD63 to immature WPBs [47].

During maturation, WPBs also acquire Rab proteins, in particular the small GTPase Rab27A, which is important for the regulation of WPBs exocytosis. Rab27A mediates WPBs anchorage to the actin cytoskeleton by cooperating with MyRIP and Myosin Va, located close to the plasma membrane [73]. WPBs exocytosis is primarily driven by increasing intracellular Ca2+ or cyclic adenosine monophosphate (cAMP) that function as second messengers, stimulating the interaction between WPBs and the plasma membrane upon ECs stimulation by various agents (e.g., thrombin, histamine, VEGF). Tethering of WPBs to the plasma membrane is mediated by Munc13‐4, which serves as a linker between the membrane and WPBs. Munc13‐4 interacts with phosphatidylinositol 4,5‐bisphosphate (PIP2)‐rich microdomains on the plasma membrane via the Annexin A2‐S100A10 complex, while simultaneously binding Rab27A on WPBs, facilitating their docking and priming for exocytosis [74]. The final steps of WPBs exocytosis involve their fusion with the plasma membrane. Specifically, the Rab27A‐Munc13‐4 complex interacts with synaptotagmin‐like protein 4a (Slp4‐a), which in turn binds SNARE proteins on both WPBs and the plasma membrane. On WPBs, the v‐SNARE proteins VAMP3 and VAMP8 facilitate vesicle docking, while on the plasma membrane, the t‐SNARE proteins STX3, STX4, and SNAP23 mediate membrane fusion, ensuring proper vesicle orientation and exocytosis [72]. Synaptotagmin 5 (SYT5) plays a regulatory role in WPBs fusion and secretion, acting as a regulator of Ca^2+^ levels by recruiting Ca^2+^‐dependent effector molecules, helping to prevent premature release of WPBs cargo [75]. Recently, STX5‐depleted ECs were shown to display significant morphological alterations in the Golgi apparatus and retention of VWF in the ER, resulting in severe defects in WPBs biogenesis and exocytosis [76]. The VWF secretion occurs through both a constitutive and a regulated pathway, each involving distinct activation mechanisms [77]. Regulated VWF secretion occurs in two phases: an initial rapid release of high‐molecular‐weight VWF upon WPBs fusion with the plasma membrane [78], followed by a delayed expulsion of approximately 20%–30% of VWF. This delayed phase is associated with the formation of an actomyosin ring around post‐fusion WPBs [79], likely contributing to the stabilization of WPBs structure and facilitating complete cargo release [80].

### 3.6 The Importance of Platelet Dense Granules in Endothelial Permeability

ECs form a barrier between vessels and tissues, regulating the flow of substances and fluid in and out of tissues. Under normal conditions, ECs adhere to each other and to the extracellular matrix (ECM) through inter‐endothelial junctions (IEJs) and integrin receptors, which provide the mechanical strength and tightness required to establish a barrier, while also enabling intercellular communications [81].

In physiological conditions, IEJs are impermeable to macromolecules. However, during pathologic phenomena, such as acute or chronic inflammation, ischemia–reperfusion, atherosclerosis, sepsis, diabetes, thermal injury, etc., mediators such as histamine, serotonin, thrombin, bradykinin, substance P, platelet‐activating factor (PAF), cytokines, growth factors, and reactive oxygen species (ROS) induce ECs retraction, which increases the intercellular spaces and subsequently the permeability to solutes and plasma proteins. Alterations of endothelial barrier function lead to abnormal extravasation of fluid and macromolecules, resulting in edema and endothelial dysfunction [82].

The barrier function of endothelium not only relies on ECs themselves but also on platelets, which turned out to be one of the main players in the regulation of vascular permeability.

In a recent study, Gupta et al. found that severe thrombocytopenia in mice (a drop of < 5% of control) was sufficient to cause a reversible increase in the permeability of capillary and post‐capillary venules [83]. Thrombocytopenia caused increased extravasation of 40‐kDa dextran from capillaries and postcapillary venules but did not affect extravasation of 70‐kDa dextran or albumin. This reduction in barrier function required more than 4 h to emerge after thrombocytopenia was established, reverting to normal as the platelet count recovered. Barrier dysfunction was observed in mice deficient in platelet‐dense granules, the machinery for dense granule secretion, glycoprotein (GP) VI, or the GPVI signaling effector phospholipase C (PLC) γ2. However, it was not observed in mice lacking α‐granules, C‐type lectin receptor‐2 (CLEC‐2), or protease‐activated receptor 4 (PAR4). In particular, BLOC‐1^−/−^ (Pallid) mice, which lack platelet‐dense granules [84], demonstrated increased extravasation of 40‐kDa dextran compared to controls. Similarly, Unc13dJinx mice [85], which possess platelet‐dense granule contents but are unable to secrete them, display impaired endothelial barrier function.

Therefore, the secretion of dense granules plays a crucial role in maintaining vascular integrity. However, it is still unknown which specific molecules within the dense granules are involved.

### 3.7 Pulmonary Fibrosis

#### 3.7.1 Molecular Pathways Involved in Pulmonary Fibrosis

Pulmonary fibrosis in HPS is mainly associated with functional defects in the BLOC‐3 and AP‐3 complexes [86].

In lungs, ECs establish a semi‐permeable barrier between the bloodstream and surrounding tissues, playing a crucial role in gas exchange, solute transport, and fluid regulation. The integrity of the pulmonary endothelial barrier is preserved through a coordinated network of receptors, signaling molecules, junctional complexes, and protein‐regulated cytoskeletal rearrangements. In conditions such as acute lung injury (ALI) or acute respiratory distress syndrome (ARDS), the loss of this barrier's integrity due to endothelial dysfunction driven by severe pulmonary inflammation or infection results in pulmonary edema and hypoxemia [87]. Pro‐inflammatory mediators, including histamine, thrombin, bradykinin, interleukin‐1β, tumor necrosis factor‐α, vascular endothelial growth factor, angiopoietin‐2, platelet‐activating factor, bacterial toxins, and ROS, induce cytoskeletal alterations, disrupt adherens junctions, and cause the detachment of vascular endothelial cadherin from the actin cytoskeleton, thereby increasing endothelial permeability.

Regarding ROS, it is hypothesized that they drive fibrotic remodeling by inducing epithelial damage, as well as promoting lung inflammation and myofibroblast activation. A study on HPS‐1 mice found increased levels of mitochondrial ROS in mice lung tissue, suggesting that an increase in ROS can be an early sign of pulmonary fibrosis [88].

Dysfunctional AT2 cells [89] promote persistent recruitment of inflammatory cells [90] and the deposition of ECM [91], leading to an irreversible modification of the lung structure, characterized by a foam‐like degeneration of AT2 cells and the presence of ceroid‐like deposits in alveolar macrophages [86]. In relation to HPS, Wang et al. demonstrated that aberrant AT2 progenitor cell differentiation and p53 pathway dysregulation can trigger the onset of fibrosis development [92]. The LBs are localized within AT2 cells, where they are involved in the synthesis and secretion of surfactant, a mixture of lipids and proteins that maintain alveolar integrity. HPACs, in particular AP‐3, are involved in the delivery to LBs of peroxiredoxin 6 (PRDX6), a soluble enzyme involved in their maturation. Thus, when AP‐3 is altered, an aberrant LROs maturation occurs, contributing to the impaired delivery of PRDX6 to LBs with the subsequent accumulation of surfactant, resulting in pulmonary damage [93]. AP‐3 modulates the signaling pathway of YAP, a key regulator of the regenerative and repair capacity of AT2 cells. Both the overactivation and the downregulation of the YAP signaling pathway can lead to the development of pulmonary fibrosis: the first leads to the dysregulation of alveolar epithelium, and the second prevents the differentiation of AT2 cells into AT1 cells, promoting the deposition of collagen and inflammation [86]. P4 ATPase ATP8A1 is an endosomal phosphatidylserine (PS) flippase, present on recycling endosomes, and activates YAP by flipping PS toward the cytoplasmic site of endosomes. AP‐3 is responsible for the transport of P4 ATPase ATP8A1 from endosomes to lamellar bodies. Therefore, a mutation of the AP‐3 complex can lead to an accumulation of PS in the cytoplasmic site of endosomes and consequently to an overactivation of the YAP pathway [94]. These mechanisms impair the maturation of lamellar bodies and may play a role in the pathogenesis of pulmonary fibrosis in HPS‐2 and HPS‐10 patients [86].

Dysfunctional AT2 cells become apoptotic due to ER stress and the recruitment of alveolar macrophages, with an excessive production of monocyte chemoattractant protein‐1 (MCP‐1) and transforming growth factor‐β (TGF‐β) [95, 96]. HPS‐2 patients show high plasma concentrations of TGF‐β1 (one of the three isoforms of the pro‐fibrotic cytokine) and pro‐inflammatory cytokine IL‐17A [97]. Dysfunctional AT2 cells and alveolar macrophages overproduce TGF‐β which binds to specific receptors, TGFBR1 and TGFBR2, and activates sequential downstream intracellular signaling through SMAD proteins, which regulate gene expression that initiates fibrosis, such as fibroblast differentiation into myofibroblasts, increased deposition of ECM components, and epithelial‐mesenchymal transition (EMT) [98]. EMT is a reversible mechanism that, if excessively activated, may establish a pro‐fibrotic environment, as it suppresses epithelial features while promoting mesenchymal markers [99]. The genotypic and phenotypic change is related to ER stress and the activation of epidermal growth factor receptor (EGF) signaling in AT2 cells [100]. In parallel, the TGF‐β/SMAD pathway also promotes the differentiation of quiescent fibroblasts into myofibroblasts, characterized by an abnormal ECM synthesis with unbalanced components. This leads to increased matrix stiffening, which plays a role in the pathogenesis of pulmonary fibrosis [101].

#### 3.7.2 Matrix Metalloproteinase Activity in the Lung Is Increased in Hermansky‐Pudlak Syndrome

Matrix metalloproteinases (MMPs) are a family of zinc‐dependent enzymes involved in numerous physiological and pathological phenomena related to ECM dynamics, such as tissue remodeling, cell migration, and angiogenesis. Additionally, MMPs influence several cell functions by modulating the activity of certain chemokines and cytokines or by directly acting on cell surface receptors, thereby initiating cell signaling [102].

Dysregulated MMP activity has been linked to the pathogenesis of numerous chronic lung diseases, including asthma, emphysema, cystic fibrosis, and idiopathic pulmonary fibrosis (IPF) [103]. Evidence suggests that various upregulated MMPs play a profibrotic or anti‐fibrotic role in IPF by contributing to abnormal tissue remodelling and also influencing the epithelial and mesenchymal cell behaviour [104]. The role of MMPs in the pathogenesis of IPF involves the promotion of epithelial‐to‐mesenchymal transition (MMP‐3 and MMP‐7); the increase of lung levels or activity of profibrotic mediators and/or the reduction of antifibrotic stimuli (MMP‐3, MMP‐7, and MMP‐8); the promotion of abnormal epithelial cell migration and other aberrant repair processes (MMP‐3 and MMP‐9); the induction of the switching of lung macrophage phenotypes from the M1 to the M2 subtypes (MMP‐10 and MMP‐28); and the promotion of fibrocyte migration (MMP‐8) [105].

Summer et al. demonstrated that mutations in two different HPS genes lead to an upregulation in the expression and activity of MMPs, similar to that shown in IPF. They found that MMP‐2 and ‐9 are increased in the lung epithelium of HPS1 and HPS2 mice and that elevated MMP levels are associated with an increase in Akt activation. Furthermore, they showed, both in mice and humans, that these changes precede the development of pulmonary fibrosis, suggesting a potential role of MMPs in priming the lung to injury in subjects with HPS [103]. Taken together, these findings unravel another pathogenic mechanism of this syndrome. They show that HPS‐related genes may play a crucial role in regulating MMPs in the lung and suggest that altered MMP expression can contribute to lung deterioration and fibrotic remodeling.

### 3.8 Inflammatory Bowel Disease (IBD)‐Like Manifestations

A subset of HPS patients can develop granulomatous colitis (GC), an intestinal inflammatory condition resembling Crohn's disease (CD) [106] determined by inappropriate gut microbiota and host immune response, leading to altered mucosal homeostasis [107]. The precise mechanism of GC pathogenesis in HPS patients is still under investigation. To date, it is known that the HPS‐1 and HPS‐4 proteins are involved in the formation of the BLOC‐3 complex [108] which functions as a GEF for Rab32/38 [109] and is involved in the regulation of cargo trafficking to LROs in specialized cell types, such as melanocytes. However, the role of Rab32/BLOC‐3 in regulating host defense against different pathogens was also demonstrated, highlighting its importance in the immune response [110]. Moreover, Cavounidis et al., revealed that HPS‐1 patients have dysfunctional macrophages resulting in an altered anti‐microbial response which may lead to chronic inflammation and epithelial barrier damage, commonly seen in these patients. Intracellularly the HPS‐1 protein is involved in phagosome maturation by lowering mTORC1 levels through Rab32, and by facilitating microbial degradation. When HPS1 is mutated, mTORC1 levels increase, altering the lipid droplet metabolism and leading to aberrant bacterial clearance and dysregulation in cytokine secretion (TNF, IL‐1, PTGS_2_, OSM). This results in an altered anti‐microbial response by tissue‐resident macrophages, leading to chronic inflammation and epithelial barrier damage [111]. Large dense‐core vesicles (LDCVs) are a type of LROs that form at the TGN and are involved in peptide secretion in various secretory cells. In the intestine, Paneth cells (PCs), which are specialized epithelial cells, release acidophilic granules containing antimicrobial peptides, contributing to the maintenance of the intestinal microbiome. A link between PCs defective function and CD pathogenesis has been suggested [112]. In PCs, bacterial products are detected by the cytoplasmic receptor Nod2, which regulates lysozyme sorting through the Nod2‐LRRK2‐Rab2a axis. Disruptions in this pathway lead to defective lysozyme trafficking and function [113]. An abnormal LDCV morphology of PCs, characterized by an increased number and size of LDCVs, was found in HPS‐1 deficient *pale ear* (ep) mice which were resulting in an alteration of the secretion of lysozyme, an antimicrobial enzyme important for gut immunity the alteration of which can increase intestinal inflammation [114]. HPS‐1 protein acts as a GEF for Rab32/38, facilitating VAMP7 recycling for LDCVs maturation. Defective recycling in HPS1‐deficient cells leads to immature LDCVs, impacting lysozyme secretion with consequent impairment of intestinal homeostasis. Although deposition of ceroid‐like pigments was found in the HPS patient's gastrointestinal tract, their accumulation in intestinal macrophages does not contribute to inflammation since some patients developed GC without the presence of ceroid pigments [115].

### 3.9 Neutropenia

The immunodeficiency and/or neutropenia seen in patients with the HPS‐2 and HPS‐10 subtypes are commonly correlated to the impairment of the AP‐3 complex, resulting in increased susceptibility to infections due to defects in several mechanisms required for a correct immune response to antigens. Antigen processing and presentation is a critical mechanism in which antigen‐presenting cells (APCs), such as dendritic cells (DCs), recognize exogenous materials (antigens), process them by the proteasome, and then load them into the MHC expressed on the cell surface. It was reported that AP‐3 is involved in the delivery of TLR to phagosomes upon antigen uptake. Thus, DCs with AP‐3 deficiency showed impaired antigen presentation to CD4+ T cells due to inefficient peptide/MHC‐II complex expression on the surface of DCs and an impaired proinflammatory response upon infection [116]. Besides AP‐3 involvement in the sorting to the phagosome of TLR9 [117], Ocwieja et al. showed that IL‐6 signaling required AP‐3, which is responsible for TLR2 intracellular trafficking. The reduced number of natural killer T (NKT) cells found in HPS‐2 and HPS‐10 patients may be explained by an impairment of glycolipid antigen presentation by APCs to NKT, which leads to persistent infections. In detail, it was demonstrated that AP‐3 is involved in the trafficking of CD1b to the lysosomes for glycolipid antigen acquisition and presentation to immune cells. Mutations in the AP‐3 complex affect the CD1b pathway, resulting in impaired antigen presentation to NKT and consequently decreased activation. This explains the recurrent bacterial infections in HPS‐2 patients. Moreover, defects in innate immunity were also detected. Circulating neutrophils contribute to a rapid immune response upon infection by phagocytosing invading pathogens and releasing enzymes stored within their granules. Neutrophil elastase (NE) is an enzyme that relies on AP‐3 for its trafficking to azurophil granules. In AP‐3 deficient neutrophils, it was observed that NE was missorted or degraded prematurely, impairing neutrophil maturation and contributing to neutropenia [118]. Similarly, NK and CTL cells showed defective cytolytic activity in HPS‐2 patients due to a defect in polarization and lytic granule content [119], highlighting an important role of HPS proteins in protein sorting and immunity.

## 4 Clinical Manifestations of Hermansky‐Pudlak Syndrome

**FIGURE 2:**
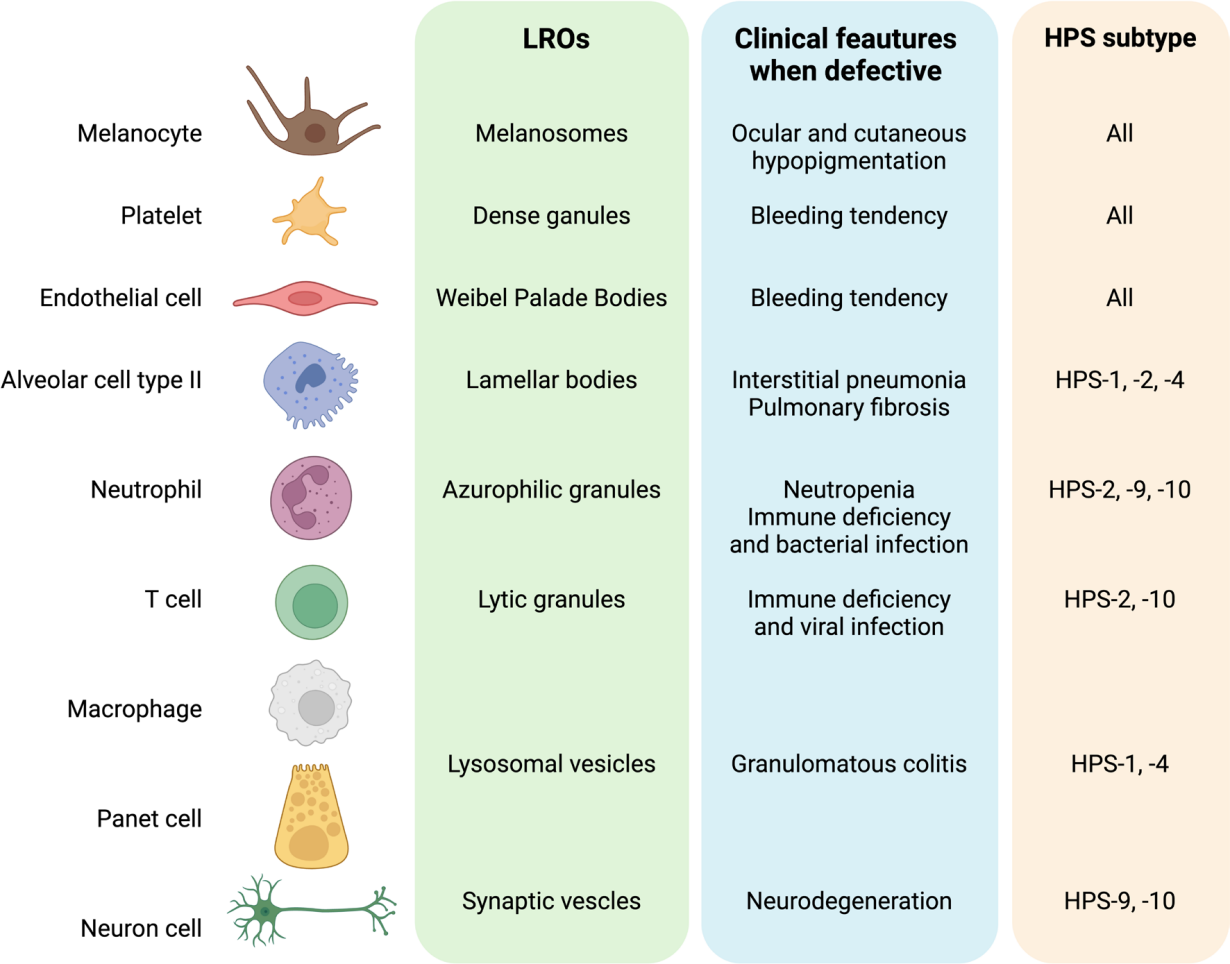
Most common clinical manifestation in HPS subtypes with associated defective LROs and cell type. Created in BioRender. https://BioRender.com/p55c817.

All HPS patients are characterized by oculocutaneous albinism (OCA) and platelet storage pool deficiency. However, HPS subtypes have different additional signs and symptoms according to the LROs complex that is mutated, as shown in Figure 2. For instance, HPS‐3, HPS‐5, and HPS‐6 have milder symptoms with respect to other HPS subtypes [120]; they do not develop the pulmonary fibrosis and granulomatous colitis that are seen in patients with defective BLOC‐3 (HPS1 and HPS4), or the neutropenia that is present in defective AP‐3 (HPS2 and HPS10).

### 4.1 Dermatologic Manifestations: Oculocutaneous Albinism

Patients with OCA have hypopigmentation due to the loss or reduction of melanin in the skin and retina. OCA patients have a high risk of developing UV‐induced skin cancers due to the reduction in photoprotection and to the increased formation of ROS from eumelanin and pheomelanin, respectively, in the skin. The more severe the hypopigmentation, the more serious foveal hypoplasia and reduction of visual acuity can be [6]. OCA is divided into syndromic and non‐syndromic; HPS patients belong to the first group. The degree of hypopigmentation in HPS patients is variable, depending on their subtype: patients with BLOC‐2 mutations (HPS3, HPS5, or HPS6) have relatively mild hypopigmentation with respect to patients with BLOC‐3 mutations (HPS1, HPS4). These clinical characteristics may be correlated with the relative role of BLOC‐2 and BLOC‐3: BLOC‐2 plays a more significant role in retinal pigment epithelium (RPE) than in neural crest‐derived melanocytes, while BLOC‐3, together with Rab32 and Rab38, is implicated in the endosomal trafficking of melanogenic enzymes in melanocytes, thus explaining the different extension of hypopigmentation between patients with BLOC‐2 and BLOC‐3 mutations [121].

### 4.2 Ocular Manifestations: Visual Impairment and Nystagmus

The lack or reduction of melanin in the retinal pigment epithelium disrupts normal retinal development and function, causing foveal hypoplasia, reduced visual acuity, and altered optic nerve wiring. Common clinical manifestations include amblyopia, photophobia, nystagmus, and strabismus, together with visual impairment. These manifestations are more severe in patients with BLOC‐2 mutations (HPS3, HPS5, or HPS6) due to the above‐described role of BLOC‐2 in the retina.

### 4.3 Platelet Dysfunction and Bleeding Tendency

Platelet δ granules store critical factors, like serotonin, calcium, and ADP, which are released during platelet activation to support aggregation. In individuals with HPS, the absence or significant reduction of these granules impairs the release of these factors, leading to impaired platelet activation and bleeding manifestations despite a normal platelet count. The haemorrhagic tendency typically manifests as easy bruising, epistaxis, gingival bleeding, and prolonged bleeding after minor injuries, surgery, delivery, or dental procedures. Women with HPS often experience menorrhagia. Gastrointestinal bleeding and bleeding of the central nervous system may also occur, but are rare.

In an international study, the median ISTH‐BAT bleeding score of HPS patients, a clinical score that measures bleeding severity, ranged from 2 to 18, with a median value of 5 (IQR 2–13), where a score > 3 in males and > 5 in females is considered pathologic [122]. Moreover, HPS patients experience a high frequency of post‐surgical bleeding, with 27.3% of them suffering hemorrhages after a surgical procedure [123]. A systematic assessment of the incidence of postpartum hemorrhage in HPS has not been carried out, but a review of the literature highlights 15 reported cases. An anti‐hemorrhagic prophylaxis, mainly consisting of platelet transfusions and 1‐deamino‐8‐d‐arginine vasopressin (DDAVP), was applied for all but 3 deliveries. Three women experienced excessive bleeding despite prophylaxis, while of the 3 subjects that did not receive prophylaxis, one did not have any bleeding, another experienced very mild bleeding, and the third experienced severe bleeding [124].

### 4.4 Pulmonary Fibrosis

Pulmonary fibrosis is one of the most severe complications and is associated with the HPS‐1, HPS‐2, and HPS‐4 subtypes. HPS1 and HPS4 subjects are more prone to develop pulmonary fibrosis during middle age, while HPS2 pulmonary fibrosis is more prevalent in children and young adults [86].

Dysfunctional alveolar epithelial cells, abnormal immune and inflammatory reactions, aberrant lung fibroblast proliferation, aberrant autophagy, metabolic derangement, and aging represent the main pathogenic causes of HPS‐PF [86]. However, the precise mechanism underlying HPS‐associated pulmonary fibrosis (HPS‐PF) is not fully understood, but it is believed to arise from the disruption of LBs, which are LROs, within type II pneumocytes that are responsible for the synthesis and storage of surfactant [8]. This disruption leads to macrophage‐driven inflammation and fibroblast proliferation, ultimately leading to the development of pulmonary fibrosis. Desroziers et al. reported the presence of giant LBs in HPS1 patients and Hps1/Hps2 mutant mice due to the abnormal accumulation of surfactants [125].

HPS‐PF shares many clinical and histological features with idiopathic pulmonary fibrosis (IPF) but differs in the age of onset and progression. While IPF typically manifests in individuals over the age of 50, HPS‐PF presents much earlier, often in patients between 30 and 40 years of age. HPS‐PF is characterized by the progressive scarring of lung tissue, leading to respiratory failure. The disease begins with exertional dyspnoea, which progressively worsens, eventually resulting in debilitating hypoxemia [126].

### 4.5 Inflammatory Bowel Disease (IBD)‐Like Manifestations

An inflammatory bowel disease resembling Crohn's disease develops in some individuals with HPS‐1 and HPS‐4, but its aetiology is poorly understood. The most common symptoms of HPS‐associated IBD are haematochezia, abdominal pain, and loose stools. Granulomatous colitis is a significant complication that can be severe, and about 15% of affected individuals may experience life‐threatening gastrointestinal bleeding, which sometimes necessitates surgical interventions such as colectomy.

The etiology of HPS‐IBD remains unknown, but a possible mechanism relies on the accumulation of ceroids in the GI tract. Ceroid lipofuscin is normally produced during aging, and it is cleared by lysosomal vesicles. Cells responsible for ceroid clearance in the GI tract are macrophages, and in HPS, macrophages accumulate ceroids, probably due to defects in lysosomal vesicle production. Ceroid accumulation may cause the disruption of macrophages that then induce an inflammatory response, a mechanism that can explain the IBD‐like manifestations in HPS patients [127].

Another hypothesized mechanism resides in the biogenesis of LDCVs in PCs, containing antimicrobial peptides. The HPS1 subunit of the BLOC‐3 complex is important for the maturation of LDCVs, as shown in HPS1‐deficient pale ear mice in which the maturation of PC LDCVs is defective, consequently affecting the secretion of antimicrobial peptides, with consequent alterations in the composition of the intestinal microbiota.

### 4.6 Neutropenia

Neutropenia is typical of HPS‐2, but it has also been described in HPS‐9 and HPS‐10, all subtypes with mutations in the subunits of the AP3 complex [128]. Neutropenia in HPS is typically chronic and can predispose affected individuals to recurrent bacterial and viral infections, such as the Epstein–Barr Virus, particularly of the respiratory tract. Infections often begin in early childhood, manifesting as recurrent otitis media, pneumonias, and skin infections. Moreover, HPS‐2 patients are more likely to develop Hodgkin lymphoma due to their neutropenia and abnormal cytotoxic activity of NKT. In the literature, two cases of HPS‐2 patients with Nodular lymphocyte‐predominant Hodgkin Lymphoma (NLPHL) [129] and one case of an HPS‐2 patient with hemophagocytic lymphohistiocytosis (HLH) [130] were also described.

## 5 Current Treatment Approaches

### 5.1 Management of Bleeding

Patients with inherited platelet disorders, including HPS, should be counseled and prophylactically treated to avoid bleeding events.

General prophylactic measures include avoiding medications that impair platelet function, such as aspirin and anticoagulants, and non‐steroidal anti‐inflammatory drugs [120], practicing regular dental hygiene, and avoiding trauma, especially in severe cases. Immunization against hepatitis A and B is crucial, as patients may require blood products. Indeed, platelet transfusions and packed red blood cells are used for surgery prophylaxis, favoring the use of HLA‐matched platelet concentrates from single donors to prevent alloimmunization [131]. Preoperative prophylaxis also includes the use of intravenous DDAVP, but it is generally recommended to have platelet concentrates ‘on standby’ for procedural surgeries [132] although, the use of platelet transfusions should be limited in consideration of the possible alloimmunization, insufficient availability of blood donors, and the high associated costs.

Transfusion of blood products is also recommended for the management of pregnant women during delivery or in case of severe bleeding episodes, particularly menorrhagia, menometrorrhagia, and post‐partum haemorrhage [131]. A review from literature reports 14 deliveries in 11 women: 6 deliveries were managed with prophylactic DDAVP (6/14, 42.9%), 50% of which showed severe maternal blood loss, 5 deliveries were managed with platelet concentrates (5/14, 35.7%) and no bleeding complications were observed; the remaining 3 deliveries (3/14, 21.4%) were not managed with any kind of prophylactic treatment since the diagnosis of HPS was not known in advance. Of these, one required red blood cell transfusions, one surgical packing, and one did not experience bleeding.

A worldwide, multicentric, retrospective study assessing the bleeding complications of surgery in patients with inherited platelet disorders, the SPATA study, enrolled 11 HPS patients who underwent 22 surgical procedures in total [123].

An analysis of the SPATA study database carried out *ad hoc* for this review shows that anti‐hemorrhagic prophylaxis was applied for 13 out of 22 procedures: for 7 procedures patients underwent platelet transfusion, for 1 the patient was administered anti‐fibrinolytic agents, DDAVP was administered to 4 patients, and the last was given activated factor VII as prophylaxis. Excessive bleeding was reported in 6 out of 22 procedures (27.3%). Three patients experienced bleeding at surgery despite anti‐hemorrhagic prophylaxis that for all the three cases consisted of platelet transfusions. In two cases, bleeding was severe, requiring platelet transfusions. When prophylaxis was not given, 3 patients out of 8 experienced excessive bleeding, which in one case was severe.

Iron deficiency correction is commonly required, especially in young women and children. In case of bleeding, topical hemostatic interventions involve thrombin‐soaked gel foam for skin wounds, electrocautery, nasal packing, and tranexamic acid‐soaked sponges. Systemic treatments include the administration of antifibrinolytic agents (e.g., aminocaproic acid, tranexamic acid) and DDAVP [131].

### 5.2 Treatments for Pulmonary Fibrosis

Management of pulmonary fibrosis involves preventive care for asymptomatic patients belonging to the HPS subtypes associated with pulmonary fibrosis, supportive care for those with symptomatic pulmonary fibrosis, and lung transplantation for patients with severe irreversible diseases. No medications are currently approved as treatment for HPS pulmonary fibrosis. Preventive care for asymptomatic individuals should include infection prophylaxis and avoidance of cigarette smoke or other pulmonary irritants. Daily exercise is recommended, and all patients with HPS‐1, HPS‐2, and HPS‐4 should be evaluated for lung function during adolescence and yearly thereafter to monitor the possible progression of the disease [126].

Patients with symptomatic pulmonary fibrosis who experience hypoxemia should be given supplemental oxygen.

As for IPF, corticosteroids have not shown clinical efficacy, so lung transplantation remains the only established therapeutic approach for the treatment of advanced, severe HPS‐PF [120]. It represents a definitive option, although the bleeding risk represents a major obstacle to surgery in patients with HPS. Extracorporeal membrane oxygenation can be chosen as a preventive therapeutic intervention in patients who are eligible for transplantation [120].

In 2014, the United States Food and Drug Administration approved two new antifibrotic drugs for IPF: pirfenidone and nintedanib. Both agents can slow down disease progression due to their anti‐inflammatory and anti‐fibrotic effects. Particularly, pirfenidone acts by inhibiting TGF‐β slowing down lung profibrotic inflammation, while nintedanib is an intracellular inhibitor targeting tyrosine kinases to suppress fibroblast proliferation [133]. A recent case report revealed the potentially beneficial effects of long‐term nintedanib administration in HPS‐1 after lung transplantation [133]. However, two clinical trials investigating pirfenidone in HPS gave inconclusive results [134] and a potential adverse effect of nintedanib is bleeding, which is of concern in patients with HPS due to their bleeding tendency [135].

A promising therapeutic strategy could be represented by the combination of these two drugs; in fact, it has been demonstrated that they exert cumulative antifibrotic effects on macrophages and fibroblasts [136].

In conclusion, further research is needed to identify additional therapeutic targets for more effective treatment strategies. Potential candidates include thromboxane A2 receptor (TBXA2R), cannabinoid receptor type 1 (CB1R), inducible nitric oxide synthase (iNOS), and galactose lectin‐3 (Gal‐3), which are implicated in inflammation, TGF‐β signaling, and fibroblast proliferation. These targets may hold promise for the treatment of HPS‐PF [86].

### 5.3 Treatments for Colitis

Given the clinical similarities, the medical approach to colitis has been modeled on the treatment recommendations for patients with ulcerative colitis and/or Crohn's disease. Topical and systemic corticosteroids, anti‐inflammatory drugs, and immune modulators have been used with variable therapeutic efficacy. Immunosuppressive therapies, such as azathioprine or methotrexate, may be prescribed for long‐term control of inflammation and to prevent recurrence. Also, aminosalicylates and antibiotics, such as metronidazole, have been explored as a first‐line therapy for mild to moderate cases [137]. Patients with severe, refractory HPS‐associated inflammatory bowel disease were treated with anti‐tumor necrosis factor (TNF) alpha drugs (e.g., adalimumab), and, more recently, with Infliximab showing prolonged clinical response to treatment. Bowel resection is a remaining option for the management of patients with severe disease unresponsive to medical therapy [138].

### 5.4 Others

Besides the management of major manifestations, previously discussed, the therapeutic approach to HPS requires multidisciplinary care by different specialists due to the complexity of the signs and symptoms characterizing the disease.

Adequate dermatologic care and the promotion of sun protection behaviors (sunscreens and protective clothing) are important to prevent the risk of UV‐associated skin damage (solar keratosis, photo‐aging of the skin, sunburn, and cutaneous malignancy) in patients with skin hypopigmentation [139].

Also, complete ophthalmologic evaluation is recommended in HPS patients since most persons with oculocutaneous albinism typically present nystagmus and/or strabismus, conditions that can be corrected by surgery in both children and adults. Immunodeficiency, due to chronic neutropenia or decreased cytotoxic activity of lymphocytes and natural killer cells [140] typical of type 2 HPS, requires treatment strategies to enhance immune function (e.g., granulocyte colony‐stimulating factor, G‐CSF) and prevent infections. Prophylactic antibiotics could be prescribed to reduce the risk of recurrent bacterial infection; neutropenia is typically responsive to G‐CSF, and, in severe cases, hematopoietic stem cell transplantation (HSCT) may be considered [128]. Neutropenia and recurrent infections were also described in one case of HPS‐9 in which a mutation in the pallidin gene, regulating the expression of lysosomal membrane proteins in NK cells, was present. In such cases, antibiotic prophylaxis is recommended. Episodes of severe hemophagocytic lymphohistiocytosis (HLH) occurred in a few *AP3B1*‐deficient subjects; in these cases, HSCT should be considered [141].

### 5.5 Limitations of Current Treatment Strategies

A major limitation of current treatments is the lack of curative approaches. Most of the available treatments aim to manage symptoms or to slow down disease progression without addressing the underlying genetic or molecular alterations. Preventive and supportive care measures are useful to improve quality of life and to reduce complications, but, although significant progress has been made in the understanding of HPS pathophysiology, the translation of these insights into effective therapies has lagged behind. This underscores the urgent need for research into transformative interventions that could stop or reverse disease progression.

Another significant concern is the side effects and the limited efficacy of current pharmacological options. For instance, a study in Puerto Rican patients reported that the correction of the bleeding time in patients following desmopressin administration was very variable, from some to no response, highlighting the importance of determining the clinical response to this drug before giving it for major bleeding episodes or surgical prophylaxis [142].

Several clinical trials have been performed to test the efficacy of pirfenidone in the treatment of HPS‐PF. Some studies showed no significant difference between the pirfenidone and placebo, showing that no sufficient data are available to conclude that pirfenidone is a beneficial treatment for HPS‐PF patients. A more recent study in a small cohort of subjects, instead, demonstrated an improvement in the pulmonary function of patients with few adverse effects, ranging from gastroesophageal reflux, mild to moderate nausea, diarrhoea, dyspepsia, and vomiting, photosensitivity rash, an elevated creatine phosphokinase, and a reversible elevation in alanine aminotransferase or aspartate aminotransferase. These outcomes suggest that the use of pirfenidone should be considered on a case‐by‐case basis for patients with HPS pulmonary fibrosis [143]. In addition, corticosteroids, used for HPS IPF and the relief of gastrointestinal symptoms, lead to long‐term complications such as osteoporosis, hypertension, and glucose intolerance. Similarly, biologic agents for colitis, while effective in most cases, carry the risk to induce fever, joint pain, and/or shortness of breath, side effects which may necessitate the discontinuation of therapy [138]. Taken together, these limitations and the variability of response to treatment highlight the importance of developing safer and more effective therapeutic modalities to manage HPS.

## 6 Future Therapeutic Approaches: Cell and Gene Therapy

Cell and gene therapy can be considered a promising approach for the treatment of HPS, due to its genetic nature. Gene therapy aims to correct genetic defects by delivering the functional form of the mutated gene into affected cells, restoring normal cellular function. Although these therapies are still in experimental stages, they hold great potential for providing long‐term benefits to HPS patients, offering a more targeted and effective treatment option compared to traditional therapies. Ikawa et al. developed a cell and gene therapy approach to introduce the functional form of *HPS1* into melanocytes through lentiviral vector (LV) transduction [144] as a proof of concept of the feasibility of the approach. By transducing human dermal melanocytes, isolated from HPS‐1 patients, with the functional form of the *HPS1* gene, the correction of the pigmentation of the cells with the restoration of BLOC3 has been demonstrated [144].

In AT2 cells, BLOC‐3 is important for maintaining the structure of lamellar bodies through the regulation of Rab38 and of surfactant homeostasis; thus, the validation of the efficiency of cell and gene therapy in HPS‐1 melanocytes can be instrumental for the treatment of pulmonary fibrosis related to HPS.

Cullinane et al. demonstrated that the reintroduction of the *HPS1* gene into lung fibroblasts isolated from HPS patients could reduce galectin‐3 levels, a pulmonary fibrosis marker, thus confirming that the reintroduction of the functional form of the gene can revert the disease phenotype [145].

However, several challenges need to be solved, such as the efficiency of lung epithelial cell transduction and the difficulty in the isolation of this type of cell, due to its poor accessibility [120]. Moreover, a large part of the studies explored the applicability of the cell and gene therapy approach to melanosomes and epithelial cells, but only little is known about the feasibility of this approach for megakaryocytes/platelets and ECs. Therefore, one of the main goals is still to progress in the therapy for HPS, possibly developing new cell and gene therapy strategies.

## 7 Challenges and Future Directions

The need for developing safer and more effective therapeutic strategies for managing HPS has already been highlighted. This, along with other challenges, remains a central focus in HPS research. The mechanisms contributing to HPS pulmonary fibrosis are not fully defined, and expanding the knowledge on the involvement of MMPs could be an important contribution. Further studies will be necessary to understand whether, for instance, dysregulation of MMPs contributes to the onset or progression of HPS lung disease, and this will ultimately be important for advancing our understanding of the disease and for laying the foundation for new and more effective treatments. Moreover, it seems that pulmonary ECs barrier disruption strongly contributes to the development of pulmonary fibrosis; therefore, understanding the mechanisms underlying pulmonary ECs barrier disruption is another crucial research goal.

Future studies assessing defects of vascular permeability in HPS are also warranted. Indeed, the increase in the permeability of capillary and post‐capillary venules in mice lacking dense granules or unable to secrete them [83] makes it conceivable to hypothesize that the same pattern could be observed in HPS patients.

All these challenges require a cell model that can allow us to study both the disease mechanism and the effectiveness of cell and gene therapy approaches.

Induced pluripotent stem cells (iPSCs) can be generated by reprogramming adult somatic cells and have the ability to differentiate into any cell type, making them an invaluable tool for regenerative medicine, disease modelling, and drug development, and they can be particularly instrumental in studying HPS. In particular, since alveolar epithelial cells (AECs) are difficult to isolate and maintain in culture, iPSC‐based models have been developed to study the mechanism involved in pulmonary fibrosis development in HPS patients. Korogi et al. reprogrammed fibroblasts isolated from HPS‐2 patients into iPSCs and subsequently corrected them through CRISPR/Cas9 technology to finally differentiate them into AECs [146]. Interestingly, HPS2 iPSC‐derived AECs recapitulated the disease phenotype, with altered surfactant secretion, and can therefore be instrumental for future studies aiming to discover new therapeutic agents for the treatment of HPS pulmonary fibrosis. Additionally, lung bud organoids (LBOs), three‐dimensional self‐organizing structures derived from stem cells that recapitulate key features of early lung development, have been obtained to further pursue the development of new therapeutic strategies and to study the pulmonary fibrosis mechanisms. Chen et al. generated LBOs from iPSCs and introduced HPS1 mutation to trigger an early‐onset form of pulmonary fibrosis with the accumulation of extracellular matrix and mesenchymal cells [147]. Similarly, Strikudis et al. generated a lung organoid from HPS1^−/−^ embryonic stem cells and, through genome‐wide expression analysis, showed that the upregulation of IL‐11 can play a role in fibrosis development [148]. Therefore, this cell model can potentially be used to recapitulate fibrotic lung disease in vitro for drug testing and to explore tailored therapeutic approaches. However, other clinical manifestations occur in HPS, such as recurrent bleeding, due to a platelet delta storage pool deficiency, neutropenia, oculocutaneous albinism, and the development of an iPSC‐based strategy to model these other aspects of the disease can be instrumental for investigating in detail the mechanisms that induce the altered biogenesis and maturation of LROs and to develop new therapeutic agents.

## Conflicts of Interest

The authors declare no conflicts of interest.
